# Supplementary figures and images for: First specific detection and validation of tomato wilt caused by Fusarium brachygibbosum using a PCR assay
Source: PeerJ. 2023 Nov 29;11:e16473. doi: 10.7717/peerj.16473 (PMC10693239; doi:10.7717/peerj.16473)

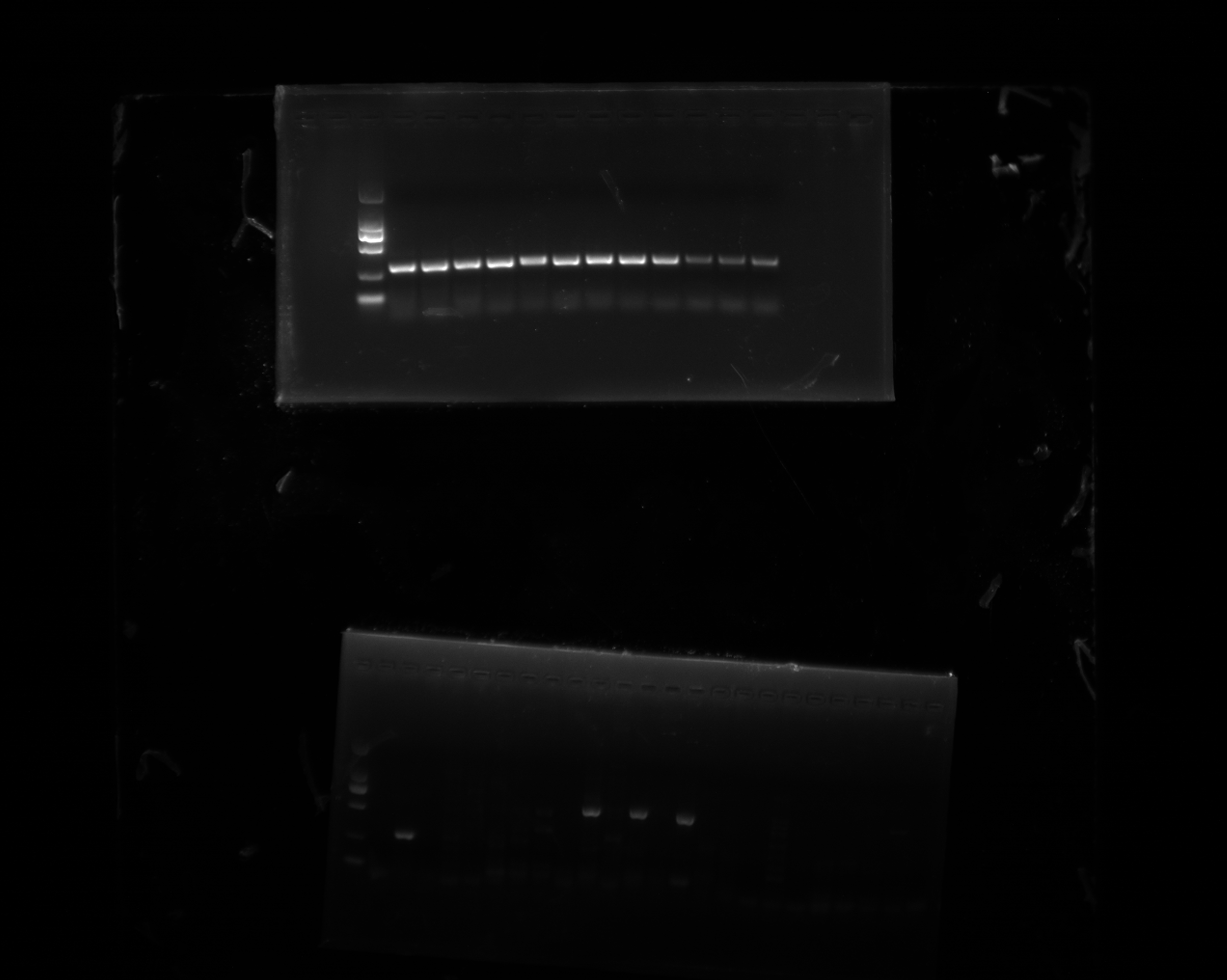


Fig.2A


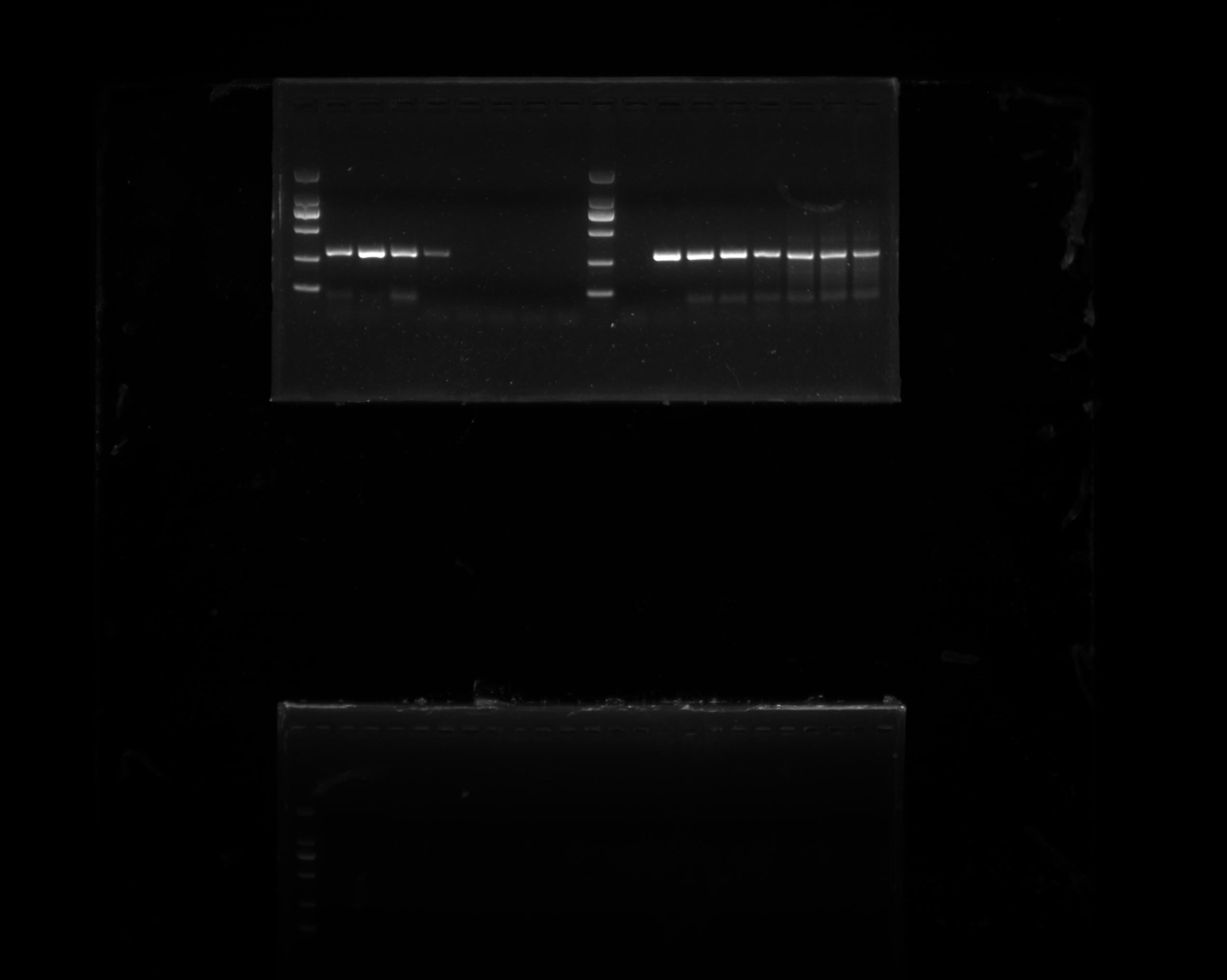


Fig.2B. Left. Fig.2C. Right


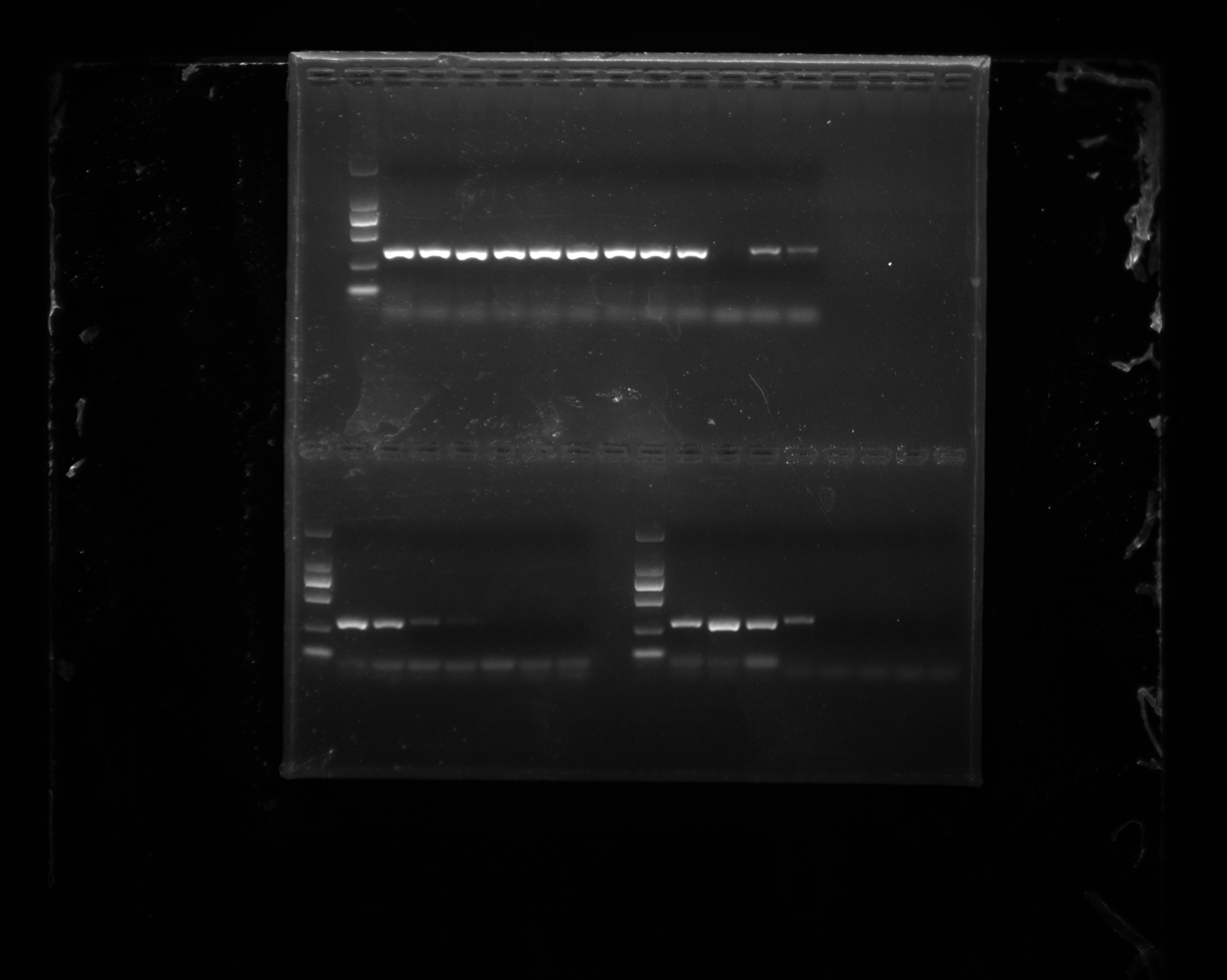


Fig.3.


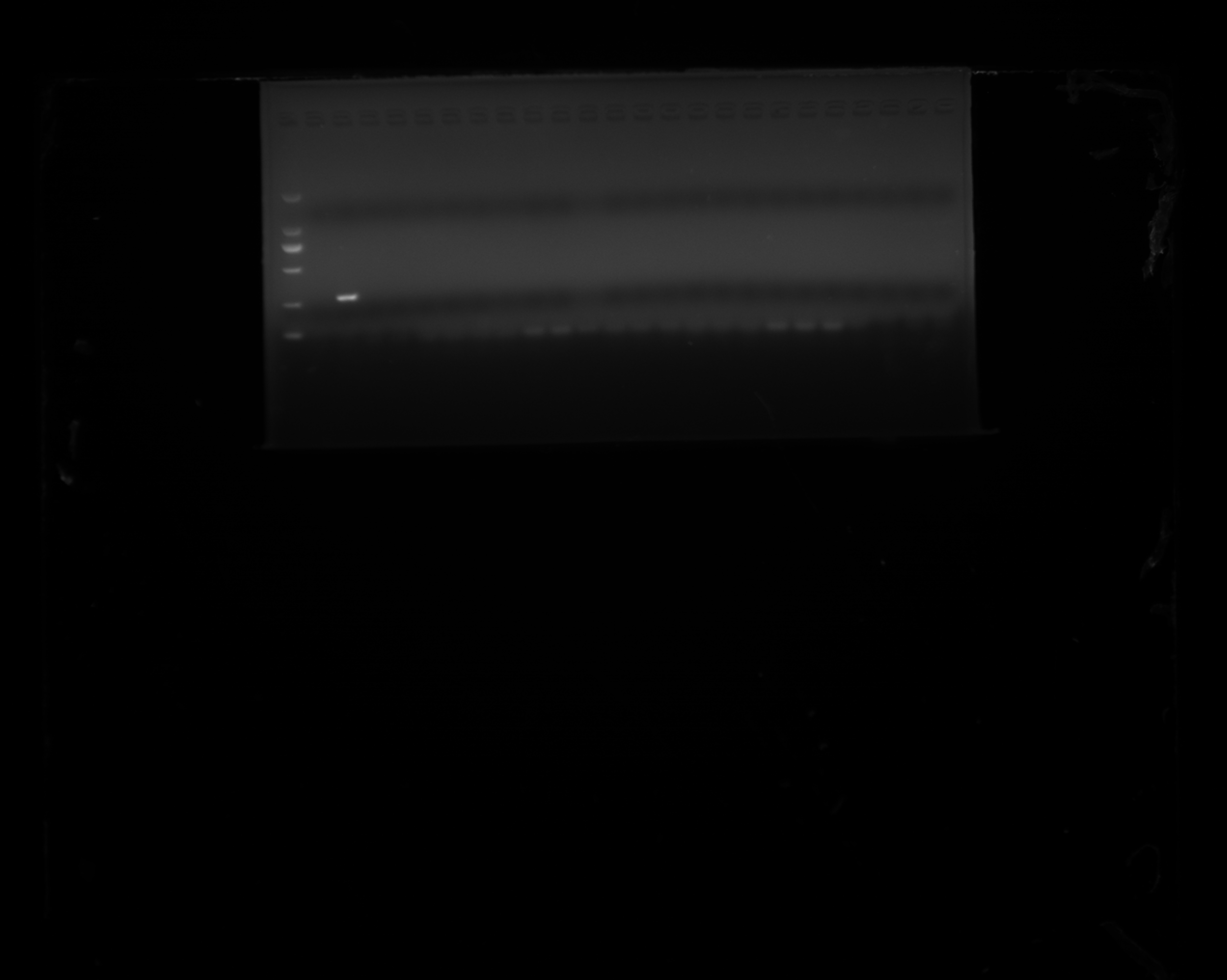


Fig.4.


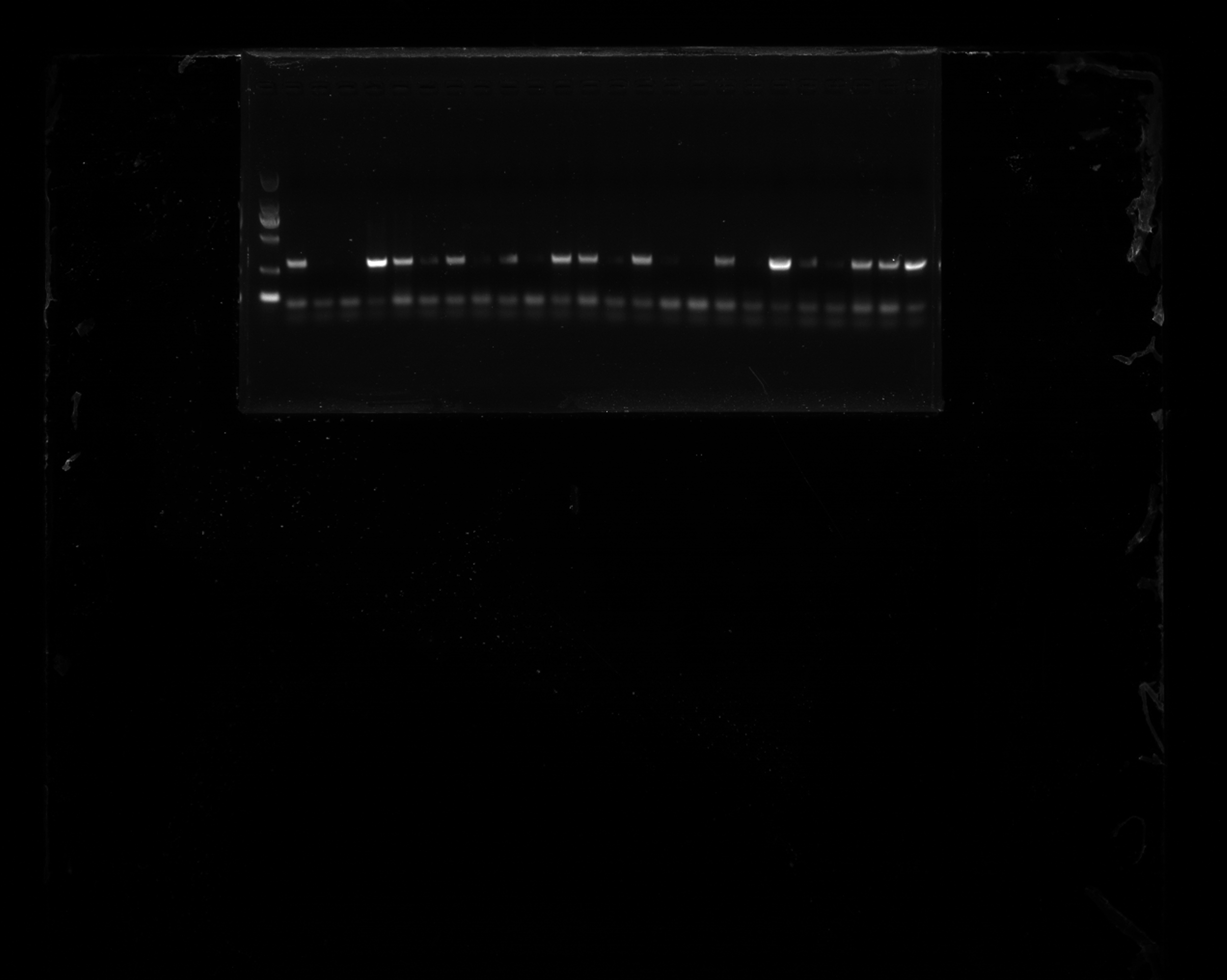


Fig.5.

Supplement: File S1 — Figure 2, 3, 4, 5 unprocessed electrophoretic gel pictures [file peerj-11-16473-s001.docx]
